# Fe(II) with Tris(1-pyrazolyl)methane Complex Increases Thermal Stability In Vitro and Activity In Vivo of the Mutant 447R Form of Mouse Tryptophan Hydroxylase 2

**DOI:** 10.3390/ijms27083411

**Published:** 2026-04-10

**Authors:** Ekatherine I. Terentieva, Polina D. Komleva, Sophia M. Antonova, Vitalii S. Moskaliuk, Nikita V. Khotskin, Maxim V. Shamshurin, Maxim N. Sokolov, Alexander V. Kulikov

**Affiliations:** 1 The Federal Research Center Institute Cytology and Genetics, Siberian Branch of Russian Academy of Sciences, Novosibirsk 630090, Russia; terentevaei@bionet.nsc.ru (E.I.T.); komleva@bionet.nsc.ru (P.D.K.); antonovasm@bionet.nsc.ru (S.M.A.); moskaliukvs@bionet.nsc.ru (V.S.M.); khotskin@bionet.nsc.ru (N.V.K.); 2 Nikolaev Institute of Inorganic Chemistry, Siberian Branch of Russian Academy of Sciences, Novosibirsk 630090, Russia; shamshurin@niic.nsc.ru (M.V.S.); caesar@niic.nsc.ru (M.N.S.)

**Keywords:** Fe(II) tris(1-pyrazolyl)methane complex, tryptophan hydroxylase 2, thermal stability, activity, brain, mice

## Abstract

Pharmacological chaperones (PCs)—small molecules that normalize the 3D structure of mutant protein molecules—are promising substances for pharmacological treatment of grave hereditary pathologies. In this study, possible chaperone-like effects of the Fe(II) with tris(1-pyrasolyl)methane complex, [Fe(TPM)_2_]Cl_2_, on the mutant 447R form of mouse tryptophan hydroxylase 2 (TPH2) in vitro and in vivo were investigated. The experiments were carried out on Balb/c mice homozygous for the mutant TPH2. This complex in concentrations of 0.01, 0.05 and 0.1 mM markedly increased the temperature (T_50_) and free energy (ΔG) of the mutant TPH2 thermal denaturation in vitro. Seven intramuscular administrations of 30 and 60 mg/kg of [Fe(TPM)_2_]Cl_2_ markedly increased the TPH2 activity in the midbrain of Balb/c mice. This increase in the TPH2 activity was not accompanied with an increase in the *Tph2* gene mRNA and TPH2 protein levels. It is the first demonstration of chaperone-like activity of [Fe(TPM)_2_]Cl_2_. This complex is a promising chemical for correction of genetic alterations in TPH2 and the associated hereditary psychic disorders.

## 1. Introduction

Some single nucleotide mutations disrupt the 3D structure of protein molecules, reducing their stability, lifetime, and, consequently, functional activity. Deficiencies in the functional activity of key proteins frequently result in severe hereditary pathologies. Pharmacological chaperones (PCs)—small molecules that normalize the 3D structure of mutant protein molecules—are promising substances for pharmacological treatment of such pathologies [1,2,3,4,5]. Understanding the molecular mechanisms of action and screening for clinically effective PCs are fundamental medical problems in modern molecular pharmacology.

Preclinical screening of PCs includes two main steps. First, the selection of chemical compounds capable of increasing the thermal stability of the studied mutant molecules in vitro. This capacity is assessed by the ability of the studied compounds to increase T_50_—the temperature at which half of the studied protein molecules are denatured [6]. It is important that the compound does not reduce the functional activity of the protein. Second, the chemicals selected at the first step and capable of increasing the T_50_ of thermal denaturation of target proteins in vitro are then administered to laboratory animals, and their ability to penetrate target organs and enhance the functional activity of the mutant proteins in vivo is studied [7].

The enzyme tryptophan hydroxylase 2 (TPH2) hydroxylates L-tryptophan to L-5-hydroxytryptophan (5-HTP), the first and key step in the synthesis of the neurotransmitter serotonin in the mammalian brain [8,9,10]. Knockout of the *Tph2* gene [11,12,13] or the inhibitor p-chlorophenylalanine [14,15] dramatically reduces the TPH2 activity in the mouse brain. Some single nucleotide mutations reducing the TPH2 activity are associated with severe psychopathologies [16,17,18,19,20]. Currently, there are no known PSs capable of correcting the pathologies caused by mutations in the *Tph2* gene.

The C1473G substitution in the *Tph2* gene resulting in the P447R substitution in the mouse TPH2 molecule leads to the 1.5-fold decrease in the enzyme activity in the mouse brain [21,22,23]. This mutation reduces T_50_ [24] and the free energy of thermal denaturation (ΔG) [25] of the enzyme, which results in decreased stability, lifetime, and activity. This mutation is common among laboratory mice [22,23] and, therefore, it is a convenient model for screening PCs for correction of pathologies associated with TPH2 deficiency.

Recently, it was shown that iron salts FeSO_4_ and FeCl_3_ increase the T_50_ and ΔG of thermal denaturation of the mutant form of TPH2 in vitro [26,27]. Moreover, the subchronic administration of the Fe(III) hydroxide dextran complex “ferrum lec” increases the activity of the mutant form of the enzyme in the mouse brain in vivo [27]. However, this increase is relatively small and does not normalize the enzyme activity to the wild-type level. Therefore, there is a need to search for other iron complexes with a more pronounced chaperone activity. A complex of Fe(II) with tris(1-pyrazolyl)methane (TPM), [Fe(TPM)_2_]Cl_2_, was selected as a potential candidate. TPM itself is widely used as a ligand for the formation of complexes with transition metal. Some of these complexes are used in medical diagnostics and oncology [28,29].

The aim of this study was to investigate the chaperone activity of the complex [Fe(TPM)_2_]Cl_2_. For this purpose, the effect of [Fe(TPM)_2_]Cl_2_ on (1) the thermal stability and activity in vitro and (2) the activity in vivo in the mouse brain of the mutant 447R form of TPH2 was examined.

## 2. Results

### 2.1. Effect of [Fe(TPM)_2_]Cl_2_ and TPM on Activity, T_50_ and ΔG of Thermal Denaturation of the 447R Form of TPH2 In Vitro

**Figure 1:**
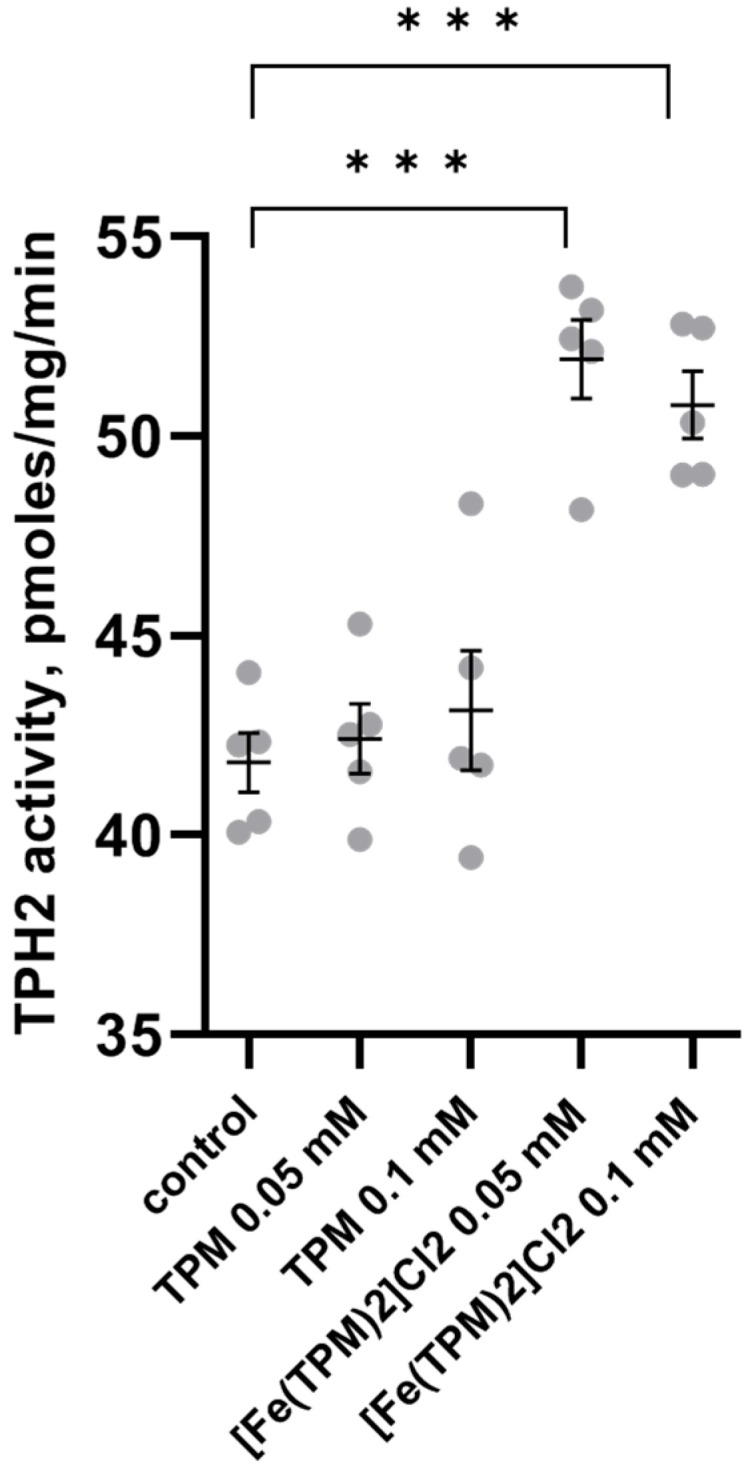
Activity of the 447R mutant form of TPH2 from the midbrain of Balb/c mice incubated with 0.05 and 0.1 mM of TPM and [Fe(TPM)_2_]Cl_2_ in vitro. Numbers of observations of TPH2 activity control, 0.05 of TPM, 0.05, 0.1 mM of [Fe(TPM)_2_]Cl_2_ *** *p* < 0.001 vs. control.

**Figure 2:**
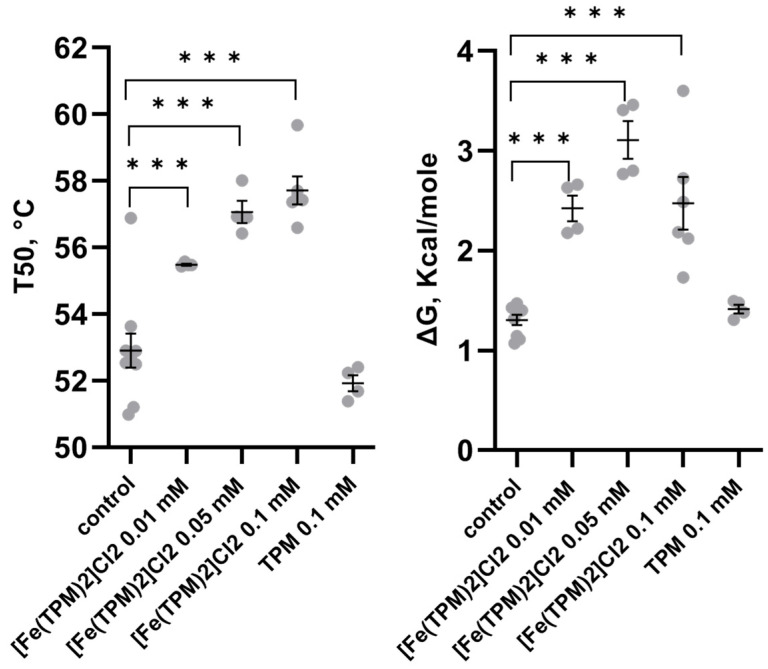
T_50_ and ΔG of thermal denaturation of the 447R mutant form of TPH2 from the midbrain of Balb/c mice incubated with 0.01, 0.05, 0.1 mM of [Fe(TPM)_2_]Cl_2_ and 0.1 mM of TPM in vitro. *** *p* < 0.001 vs. control.

[Fe(TPM)_2_]Cl_2_ markedly increased activity (F(4,20) = 23.06, *p* < 0.001), T_50_ (F(4,23) = 26.71, *p* < 0.001) and ΔG (F(4,22) = 23.8, *p* < 0.001) in vitro. Incubation of the mutant TPH2 form with two high concentrations of 0.05 and 0.1 mM of this complex markedly increased its activity (Figure 1). Preincubation of the mutant TPH2 with all three concentrations of [Fe(TPM)_2_]Cl_2_ increased T_50_ and ΔG of the enzyme thermal denaturation (Figure 2). However, TPM did not modify the activity as well as T_50_ and ΔG of thermal denaturation of the mutant enzyme (Figure 1 and Figure 2).

### 2.2. Alterations in Body Mass, Iron, 5-HT, 5-HIAA Levels and TPH2 Activity in the Brain of Balb/c Mice After Seven Intramuscular Administrations of 30 and 60 mg/kg of [Fe(TPM)_2_]Cl_2_

One mice in the group treated with 60 mg/kg of the complex died during the treatment (Fisher exact, *p* = 1.0). The remaining 23 mice were alive.

**Figure 3:**
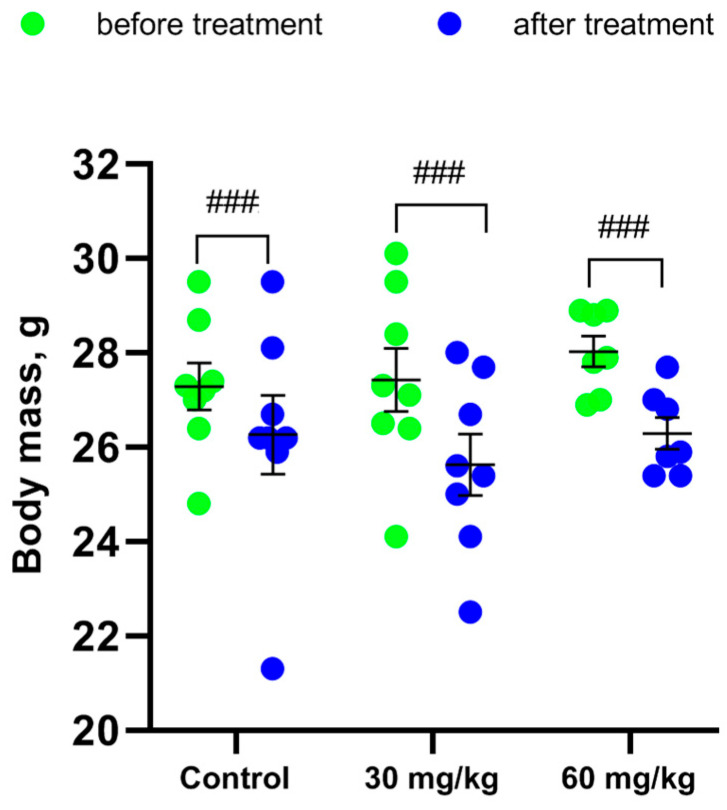
Body mass of Balb/c mice before and after 7 im injections of saline, 30 and 60 mg/kg of [Fe(TPM)_2_]Cl_2_. ### *p* < 0.001 vs. the same mice before injections.

Repeated measure ANOVA did not reveal the effect of the “Drug” factor (F(2,20) < 1) and the “Drug” X “Time” interaction (F(2,20) = 1.03, *p* = 0.38) on body mass. At the same time, an effect of the “Time” factor on body mass was shown (F(1,20) = 45.27, *p* < 0.001). The administration of two doses of [Fe(TPM)_2_]Cl_2_ during 7 successive days did not alter the body mass in mice compared to the saline-treated control (Figure 2). But the procedure of the intramuscular injection for 7 successive days itself dramatically decreased the body mass in all groups of Balb/c mice (Figure 3).

**Figure 4:**
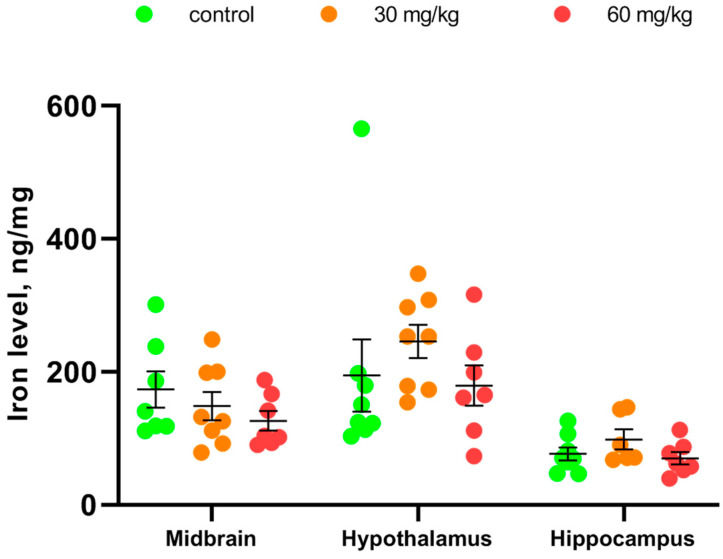
Iron concentrations in the midbrain, hypothalamus and hippocampus in Balb/c mice after 7 im injections of saline, 30 and 60 mg/kg of [Fe(TPM)_2_]Cl_2_.

Repeated intramuscular treatment with [Fe(TPM)_2_]Cl_2_ during 7 successive days did not alter iron levels in the midbrain (F(2.19) = 1.12, *p* = 0.35), hypothalamus (F(2.20) < 1) and hippocampus (F(2.18) = 1.6, *p* = 0.23) (Figure 4).

**Figure 5:**
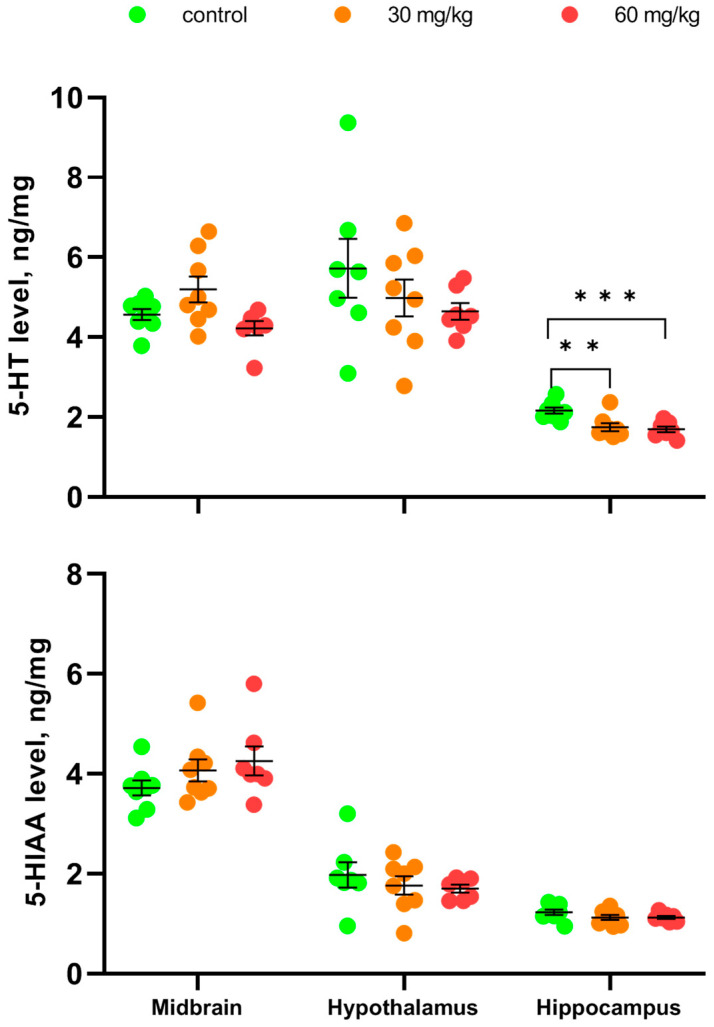
5-HT and 5-HIAA levels in the midbrain, hypothalamus and hippocampus after 7 im injections of saline, 30 and 60 mg/kg of [Fe(TPM)_2_]Cl_2_. ** *p* < 0.01, *** *p* < 0.001 vs. saline-treated.

Repeated intramuscular treatment with [Fe(TPM)_2_]Cl_2_ during 7 successive days did not alter 5-HT and 5-HIAA levels in the midbrain (5-HT, F(2,20) = 3.35, *p* = 0.056; 5-HIAA, F(2,20) = 1.5, *p* = 0.25) and hypothalamus (5-HT, F(2,20) = 1.1, *p* = 0.35; 5-HIAA, F(2,20) < 1) (Figure 5). At the same time, both doses of [Fe(TPM)_2_]Cl_2_ decreased the 5-HT level (F(2,20) = 9.46, *p* = 0.0012), but not the 5-HIAA level (F(2,20) = 1.63, *p* = 0.22) in the hippocampus (Figure 5).

**Figure 6:**
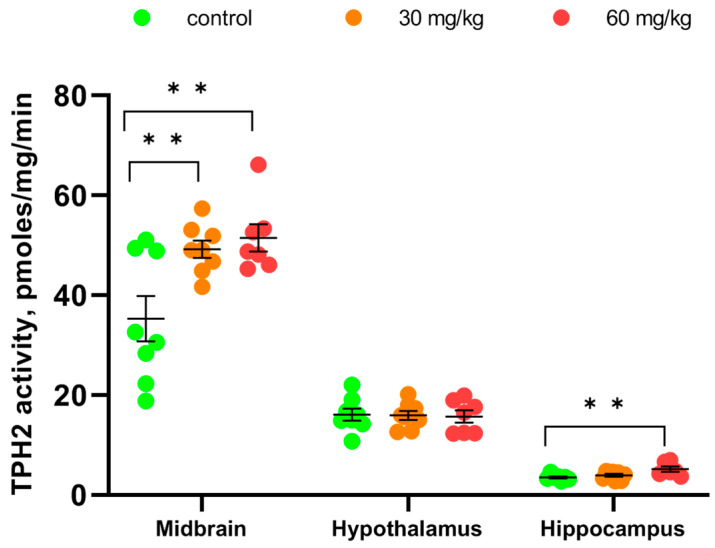
TPH2 activity in the midbrain, hippocampus and hypothalamus after 7 im injections of saline, 30 and 60 mg/kg of [Fe(TPM)_2_]Cl_2_. ** *p* < 0.01 vs. saline-treated.

Repeated administrations for 7 days of both doses of [Fe(TPM)_2_]Cl_2_ increased the TPH2 activity in the midbrain approximately 1.5 times over the control value (F(2,20) = 7.34, *p* = 0.004) (Figure 6). Treatment with high doses of [Fe(TPM)_2_]Cl_2_ increased the TPH2 activity in the hippocampus (F(2,20) = 5.53, *p* = 0.013) (Figure 6). No dose of [Fe(TPM)_2_]Cl_2_ altered the TPH2 activity in the hypothalamus (F(2,20) < 1) (Figure 6).

**Figure 7:**
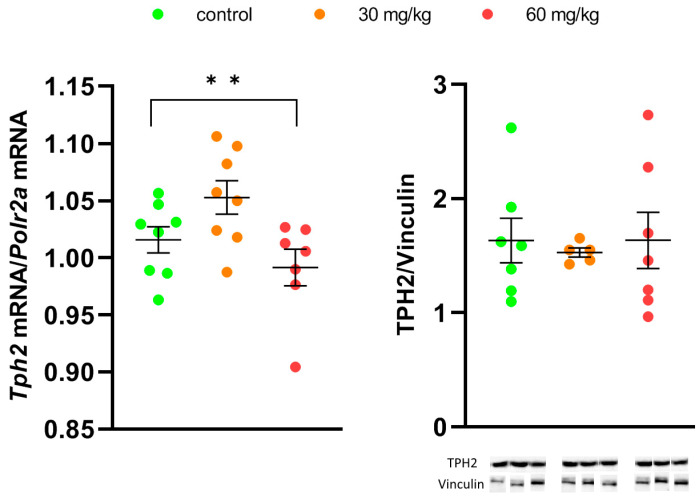
Level of *Tph2* gene mRNA and TPH2 protein in the midbrain after 7 im injections of saline, 30 and 60 mg/kg of [Fe(TPM)_2_]Cl_2_. ** *p* < 0.01 vs. control.

Repeated treatment for 7 days with high dose of [Fe(TPM)_2_]Cl_2_ decreased the *Tph2* gene mRNA level in the midbrain compared to the saline-treated value (F(2,20) = 5.22, *p* = 0.016) (Figure 7). At the same time, treatment with both doses of [Fe(TPM)_2_]Cl_2_ did not alter the density of the TPH2 protein in the midbrain (F(2,18) < 1, Supplement S1A,B) (Figure 7).

## 3. Discussion

The aim of this study was to investigate the possible chaperone activity of the complex [Fe(TPM)_2_]Cl_2_. An ideal PC should (1) increase the thermal stability of protein under study without any negative effect on the protein activity in vitro, (2) penetrate into target tissue and increase the protein functional activity in this tissue in vivo and (3) produce no negative effect on the organism. That is why the preclinical test of a chemical as a potential PC includes investigations of its effects on the target protein activity and thermal stability in vitro as well as in the organism (in vivo) [30]. Here we followed this schema.

First, the effect of the complex [Fe(TPM)_2_]Cl_2_ and the ligand TPM on the in vitro activity and thermal stability of the mutant TPH2 was investigated. Usually, the effect of a chemical on thermal stability is assessed by its ability to increase T_50_—the temperature at which half of the studied protein molecules is denatured [6]. An increase in the ΔG of thermal denaturation is an additional index of the chemical’s chaperone activity [26].

All used concentrations of [Fe(TPM)_2_]Cl_2_ increased both T_50_ and ΔG of the mutant 447R form of TPH2 in vitro. Moreover, this complex did not decrease, but its two concentrations even increased the TPH2 activity in vitro. Thus, [Fe(TPM)_2_]Cl_2_ successfully passed the first in vitro test for its chaperone activity. It should be noted that this complex causes similar increases in T_50_ and ΔG of the mutant TPH2 as F(II) iron does (see [26,27]). At the same time, the ligand TPM itself does not alter the activity, T_50_ and ΔG of thermal denaturation of the mutant TPH2 in vitro.

What is a possible molecular mechanism of this positive effect of [Fe(TPM)_2_]Cl_2_ on the mutant TPH2? The stability of [Fe(TPM)_2_]^2+^ ion is relatively low [31] and in an aqueous solution at pH 7.6, it easily dissociates into two TPM molecules and one Fe(II) ion (pK ≈ 8.62). The latter penetrates into the enzyme catalytic center and forms the dative bonds with two NE2 atoms of two histidine (H316 and H321) and one OE1 atom of glutamate (E361) residues. In the enzyme catalytic center, the Fe(II) ion seems to perform two functions: (1) catalytic function participating in the reaction of L-tryptophan hydroxylation [10] and (2) chaperone-like function stabilizing its spatial structure. The observed increases in the activity and thermal stability of the mutant TPH2 reflect respectively the catalytic and chaperone-like functions of [Fe(TPM)_2_]Cl_2_.

Since [Fe(TPM)_2_]Cl_2_ stabilizes the mutant TPH2 molecule (or at least its catalytic center) in vitro, it could be expected that treatment with this complex in vivo would normalize the mutant enzyme activity in the mouse brain. It was hypothesized that this complex administrated peripherally penetrates into the brain 5-HT neurons, accumulates there and stabilizes newly synthesized TPH2 molecules. The half-life span of TPH2 in the rodent brain is about 48 h [32,33]. Therefore, at least 4 days of treatment are needed to detect an alteration in the TPH2 activity. In the present study the complex was administered for 7 successive days. This treatment period was selected as a compromise between the time need to stabilize synthesized TPH2 and to minimize the stress caused by the injections. In the pilot experiment we showed that the doses of 30 and 60 mg/kg of [Fe(TPM)_2_]Cl_2_ did not produce any acute toxic effect on mice. These doses correspond to 3 and 6 mg/kg of iron, respectively and about 0.05 and 0.1 mM of iron—the concentrations that caused the maximal stabilization of TPH2 in vitro. Intraperitoneal administration is the most frequently used for acute and repeated treatment. However, in our pilot experiment we did not observe any effect of the repeated intraperitoneal administration on the TPH2 activity (Supplement S2). That is why an intramuscular instead of an intraperitoneal administration was chosen in the present study.

Although the treatment for 7 successive days with 60 mg/kg of [Fe(TPM)_2_]Cl_2_ killed one mouse of eight, this result was not statistically significant and did not indicate any acute toxicity of the chemical. Moreover, we did not observe any effect of the repeated treatment with the complex on body mass compared to the treatment with saline (no effect of the “Drug” factor). At the same time, the procedure of intramuscular injection itself is stressful and it similarly decreases body mass in the complex and saline-treated mice (significant effect of the “Time” factor). Treatment with both doses of [Fe(TPM)_2_]Cl_2_ for 7 successive days increased the TPH2 activity in the midbrain approximately 1.5 times compared to the control value. The activity of the mutant TPH2 in this structure became similar to the wild-type enzyme. This normalization of the mutant TPH2 activity did not result from any increase in the *Tph2* gene or TPH2 protein expression. Moreover, the high dose of [Fe(TPM)_2_]Cl_2_ even decreased the *Tph2* gene expression, maybe due to a moderate toxic effect of this chemical on the 5-HT neurons.

A high dose of [Fe(TPM)_2_]Cl_2_ also increased the TPH2 activity in the hippocampus, but not in the hypothalamus. These results do not seem unexpected since some time is needed to transport corrected mutant TPH2 molecules from 5-HT neurons cell bodies in the midbrain where they are synthesized to their terminals in the hippocampus and hypothalamus.

No association between the alterations in the TPH2 activity and 5-HT and 5-HIAA levels in the brain of mice treated with [Fe(TPM)_2_]Cl_2_ was observed. In the midbrain this treatment markedly increases TPH2, but fails to alter 5-HT and 5-HIAA levels. In the hippocampus this treatment increased the TPH2 activity, but even decreased the 5-HT level. This result seems paradoxical. However, a moderate alteration of the TPH2 activity caused by C1473G mutation does not alter the brain neurotransmitter level [34,35]. Therefore, the moderate increase in the TPH2 activity induced by [Fe(TPM)_2_]Cl_2_ treatment is not sufficient for a detectable increase in the brain’s 5-HT level. The observed decrease in 5-HT in the hippocampus of mice treated with a high dose of [Fe(TPM)_2_]Cl_2_ could result from a moderate toxic effect of this chemical on the 5-HT endings in this structure.

Now there is no direct evidence that [Fe(TPM)_2_]Cl_2_ penetrates into the brain. Now we cannot extract this complex from the brain and assay its concentration in brain samples. We also cannot detect an expected increase in iron concentration in the brain of mice treated with this complex. Nevertheless, the observed increase in the TPH2 activity in the midbrain and hippocampus of [Fe(TPM)_2_]Cl_2_ treated mice is indirect evidence that this complex penetrates into the brain’s 5-HT neurons.

Intramuscular administration of the dextran complex of hydroxide Fe(III), “ferrum lec”, in a dose of 30 mg of iron per kg of body mass for 7 successive days does not increase the mutant TPH2 activity in the midbrain of Balb/c mice. However, combined intramuscular administration of this dose of “ferrum lec” together with vitamins B_1_ and B_12_ for 7 days increased the TPH2 activity in the midbrain by 27% (see [26]). In the present study intramuscular administration for 7 days of [Fe(TPM)_2_]Cl_2_ in a dose corresponding to 3 and 6 mg of iron per kg of body mass increases TPH2 in the midbrain of Balb/c mice by 40 and 46%, respectively. Therefore, the ability of [Fe(TPM)_2_]Cl_2_ to normalize the activity of mutant TPH2 markedly exceeds that of “ferrum lec”. Iron overload is very toxic [36,37,38,39] and a prolonged treatment with “ferrum lec” will increase iron concentration in the organism above the norm that can induce multiple organ disturbances. Treatment with [Fe(TPM)_2_]Cl_2_ avoids the risk of iron toxicity.

TPH2 is the key and rate-limiting enzyme of 5-HT synthesis in the mammalian brain [8,9,10]. Its hereditary deficiency increases the risk of some grave psychopathologies [16,17,18,19,20]. Moreover, its deficiency seems to increase resistance to selective serotonin reuptake inhibitors (SSRIs) [40,41]. Until now several effective TPH2 inhibitors have been developed [23]. At the same time, there are no TPH2 activators. The natural cofactor of hydroxylase of aromatic amino acids, tetrahydrobiopterin (sapropterin), increases in vitro the thermal stability of phenylalanine [6,30,42,43], tyrosine [30,44,45] and tryptophan [24,30,44,45] hydroxylases. This chemical seems to be effective in the normalization of phenylalanine hydroxylase deficiency in the blood and liver. There are publications that report that sapropterin and its precursor, sepiapterin, are effective for therapy of phenylketonuria [46,47,48,49,50]. At the same time, this compound does not normalize deficiency of TPH2 in the brain. We showed that tetrahydrobiopterin injected intraperitoneally did not penetrate into the brain and 14 intraperitoneal injections of 48.3 mg/kg of this drug did not alter the TPH2 activity in the brain of Balb/c mice [24]. Thony and coauthors [7] orally treated young C57BL/6 mice for 5 weeks with 20 and 100 mg/kg of sapropterin and found an increase in the TH activity in the whole brain in mice treated with 100 mg/kg of the drug. However, these results are not clear, since the control values of the TH activity in the experiments with 20 and 100 mg/kg of sapropterin differed approximately 2.5 times (see ref. [7]).

In the present study, for the first time it was shown that [Fe(TPM)_2_]Cl_2_ increased (1) thermal stability in vitro and (2) TPH2 activity in the brain in vivo. In other words, this complex behaved like a typical pharmacological chaperone. It is more effective than “ferrum lec” in the treatment of TPH2 deficiency. It is important to emphasize that treatment with [Fe(TPM)_2_]Cl_2_ delivers less iron into the organism compared to “ferrum lec”. At the same time, it cannot be ruled out that [Fe(TPM)_2_]Cl_2_ may have a moderate toxic effect in addition to its chaperone-like effect and special study is needed to evaluate its toxic effect.

Pharmacological chaperones (PCs) are small molecules that restore the functional folding of mutant protein molecules [1,2,3,4,5]. Since a direct experimental visualization of the effect of PCs on the 3D structure of protein molecules is technically impossible, the increase in thermal stability is the main indirect index of the chaperone-like function of a chemical [7,30,44,45]. In this regard, it is more correct to speak about a chaperone-like effect of [Fe(TPM)_2_]Cl_2_.

The present study is the first and necessary step of preclinical screening for [Fe(TPM)_2_]Cl_2_’s ability to correct hereditary deficiency of TPH2 and associated disorders. The second stage must include the study of its positive and adverse negative pharmacological effects.

## 4. Materials and Methods

### 4.1. Animals

All experiments were carried out on 12-month-old males of Balb/c mice (n = 34, body mass 27.6 ± 0.3 g). During the experiments, the animals had specific pathogens-free state, were kept separately in cages (Optimice, Animal Care Systems, Centennial, CO, USA) at temperature 24 ± 2 °C, humidity 45–50%, and artificial 14:10 (light:dark) photoperiod with daybreak and sunset at 01:00 and 15:00, respectively, fed with sterile food and water ad libitum.

### 4.2. Chemicals

**Figure 8:**
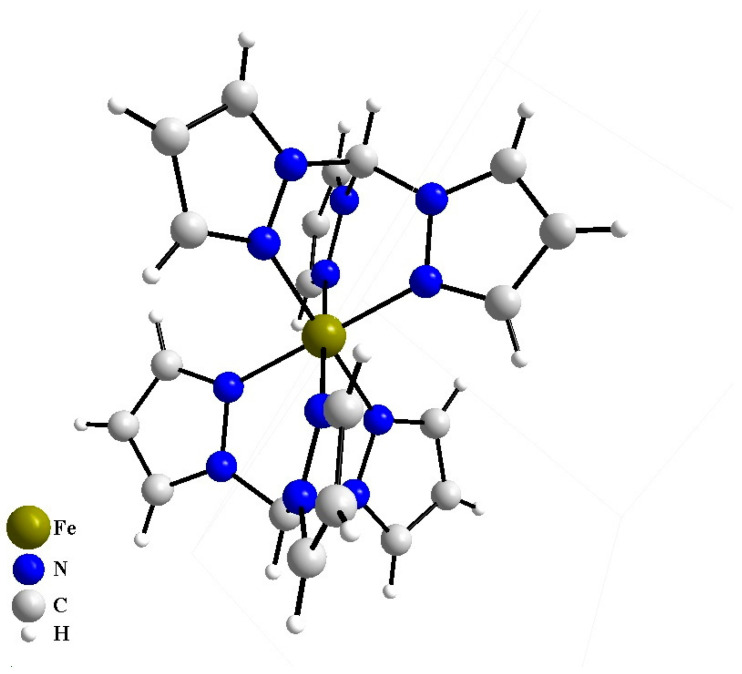
The crystal structure of [Fe(TPM)_2_]^2+^ complex cation. The complex has a distorted octahedral structure of the coordination polyhedron and the FeN6 unit.

[Fe(TPM)_2_]Cl_2_·2H_2_O was synthesized in the Nikolaev Institute of Inorganic Chemistry (Novosibirsk) according to the published protocol [51]. Twenty mL of an ethanolic solution of tris(1-pyrazolyl)methane (0.4 g, 1.86 mmol) (Sigma-Aldrich, Darmstadt, Germany) were instantly added to 15 mL of an aqueous solution of FeCl_2_·4H_2_O (0.134 g, 0.93 mmol) (Sigma-Aldrich, Darmstadt, Germany), giving a crimson-red solution. The mixture was stirred for 0.5 h under ambient conditions. Slow evaporation gave a purple crystalline product which was filtered off, washed with hexane and dried. Elemental analysis was carried out by the analytical service of the Nikolaev Institute of Inorganic Chemistry (Novosibirsk) on a Euro EA 3000 spectrometer (EuroVector Srl, Redavalle, Italy). Mass spectra were recorded on an Agilent 6130 Quadrupole MS (Agilent Technologies, Santa Clara, CA, USA). Powder X-ray diffraction (PXRD) analysis was performed at room temperature on a Shimadzu XRD-7000 diffractometer (Shimadzu Corporation, Kyoto, Japan). Yield ~83%. Anal. Calcd for C_20_H_24_N_12_O_2_FeCl_2_: C, 40.6; H, 4.1; N, 28.4. Found: C, 40.7; H, 4.1; N, 26.9. ESI-MS (*m*/*z*): 556.13 ({[Fe(TPM)_2_]Cl·2H_2_O}+), 645.1 ({[Fe(TPM)_2_]Cl_2_·5H_2_O}+). Powder pattern shows single-phase [Fe(TPM)_2_]Cl_2_·2H_2_O. The crystal structure of the complex cation is depicted in Figure 8.

In the in vitro experiment (experiment 1) 25 mM aqueous stock solutions of TPM and [Fe(TPM)_2_]Cl_2_·2H_2_O were used. In the in vivo experiment (experiment 2), 30 and 60 mg of (corresponding 3 and 6 mg of iron) [Fe(TPM)_2_]Cl_2_·2H_2_O were dissolved in 2 mL of sterile saline and then injected intramuscularly in 2 μL/g volume of body mass.

### 4.3. Experiments

Experiment 1. Effect of TPM and [Fe(TPM)_2_]Cl_2_·2H_2_O on activity and thermal stability of the mutant 447R form of TPH2 in vitro. Ten Balb/c males were euthanized with CO_2_, decapitated, and each of their 10 midbrains was homogenized in 500 μL of cold 50 mM Tris HCl buffer, pH 7.6, containing 1 mM of dithiothreitol (Sigma-Aldrich, Darmstadt, Germany), spun for 15 min at 24,700 rpm (+4 °C). The clear supernatants were pulled, aliquoted by 300 μL and stored at −80 °C as the source of the mutant TPH2 for studying the effects of various concentrations of TPM and [Fe(TPM)_2_]Cl_2_·2H_2_O on the activity and thermodynamic characters of thermal denaturation in vitro.

**Figure 9:**
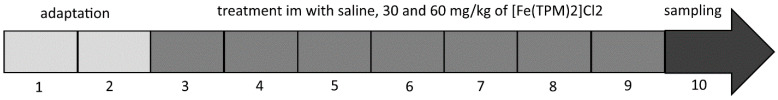
Timeline of experiment 2.

Experiment 2. Effect of repeated intramuscular treatment with 30 and 60 mg/kg of [Fe(TPM)_2_]Cl_2_·2H_2_O on the 5-HT, 5-HIAA levels and the mutant TPH2 in the brain of Balb/b in vivo. Twenty-four Balb/c males were divided into 3 groups: (1) control (n = 8), (2) 30 mg/kg (n = 8) and (3) 60 mg/kg (n = 8) of [Fe(TPM)_2_]Cl_2_·2H_2_O. Then, 30 and 60 mg of the complex were dissolved in 2 mL of sterile saline and injected into the hind leg muscle in 2 μL volume per g of body mass for 7 successive days. This corresponds to daily doses of 30 and 60 mg/kg, respectively. The controls were injected with saline for 7 successive days. On the 8th day the animals were euthanized with CO_2_, decapitated, and their hypothalamuses, hippocampus and midbrains were removed, frozen with liquid nitrogen and stored at −80 °C, Figure 9).

### 4.4. Sample Preparation

The hypothalamus, hippocampus and midbrain were homogenized respectively in 250, 300 and 400 μL of cold 50 mM Tris HCl, pH 7.6 containing 1 mM dithiotreitol. (1) Aliquot of 50 μL of the homogenate was mixed with 150 μL of 0.6 M HClO_4_ for 5-HT and 5-HIAA extraction. (2) Aliquot of 50 μL of the homogenate was mixed with 250 μL of Iron-Agate lysis buffer (Agate-Med, Moscow, Russia) for iron extraction. (3) Aliquot of 100 μL of homogenate from the midbrain was mixed with 1 mL of extractRNA reactive (Eurogene, Moscow, Russia) for total RNA extraction. (4) The rest of homogenate was spun for 15 min at 12,700 rpm (4 °C) and the clear supernatant was transferred into a clear tube and stored at −80 °C until the TPH2 activity assay.

### 4.5. Assay of Total Iron Levels

The mix of tissue homogenate (50 μL) with 250 μL of lysis buffer (2) was incubated for 30 min at room temperature, spun for 15 min at 12,700 rpm (4 °C), and 240 μL of clear supernatant was transferred into Costar 96 well plate (Corning Incorporation, Kennebunk, ME, USA) and total iron concentration was assayed according to the manufacturer’s protocol (Iron-Agate, Agate-Med, Moscow, Russia). The concentration of Fe-ferrozine complex was measured spectophotometrically at 562 nm (Multiscun GO, Thermo Fisher Scientific, Waltham, MA, USA) and expressed in ng of iron per mg of protein assay by Bradford method.

### 4.6. Assay of 5-HT and 5-HIAA Levels

The mix of tissue homogenate with 150 μL of 0.6 M HClO_4_ (1) was spun for 15 min at 12,700 rpm (4 °C), and 160 μL of clear supernatant was transferred into chromatographic vial, diluted with 160 μL of ultrapure water. 5-HT and 5-HIAA concentrations were assayed using HPLC (Shimadzu Corporation, Kyoto, Japan) with electrochemical detection DECADE II™ (Antec, Alphen aan den Rijn, The Netherlands) and expressed in ng per mg of protein as described earlier [24]. The pellet was dissolved in 0.1 M NaOH for protein concentration assay by Bradford method as described earlier [24]. Data are presented as the means of three replications.

### 4.7. Assay of the TPH Activity

Aliquot of 15 μL of clear supernatant (3) was incubated for 15 min at 37 °C with 0.4 mM of L-tryptophan (Sigma-Aldrich, Darmstadt, Germany) and 0.3 mM of tetrahydrobiopterin (Sigma-Aldrich, Darmstadt, Germany) in the final volume of 25 μL. Synthesized 5-HTP was quantified with HPLC as described earlier and expressed in pmoles 5-HTP formed per minute per mg of protein measured according to the Bradford method [26]. Data are presented as the means of three replications.

### 4.8. Assay of TPM and [Fe(TPM)_2_]Cl_2_·2H_2_O on TPH2 Thermal Stability In Vitro

Aliquots of 10 μL of clear supernatant (see Section 4.3, experiment 1) were mixed with 5 μL of 50 mM Tris HCl buffer (pH 7.6) containing 1 mM of dithiothreitol or 0.03, 0.15, 0.3 mM of [Fe(TPM)_2_]Cl_2_·2H_2_O (the final concentrations were 0.01, 0.05 and 0.1 mM or 0.3 mM) or TPM (the final concentration was 0.1 mM) solutions in this buffer and heated for 2 min at 48, 50, 52, 54. 56, 58, 60, 62 and 64 °C using a dry block thermostat TDB-120 (Biosan, Riga, Latvia) and then cooled down in ice. The control tubes were not heated. Then, 10 μL of the mix of L-tryptophan (0.75 mM), tetrahydrobiopterine (0.75 mM), m-hydroxybenzylhydrazine (0.75 mM) and catalase (5 U) in 50 mM Tris HCl buffer, pH 7.6, with 1 mM of dithiothreitol (the final concentrations of these chemicals were 0.3, 0.3 and 0.3 mM, respectively) was added, and the amount of synthesized 5-HTP was assayed using HPLC after a 15 min incubation at 37 °C (see Section 4.6). Five groups of thermal curves were formed: (1) control (n = 9), (2) with 0.01 mM of [Fe(TPM)_2_]Cl_2_·2H_2_O (n = 4), (3) with 0.05 mM of [Fe(TPM)_2_]Cl_2_·2H_2_O (n = 4), (4) with 0.1 mM of [Fe(TPM)_2_]Cl_2_ (n = 6)·2H_2_O and (5) with 0.1 mM of TPM (n = 4). The linear parts of these curves were used to calculate T_50_, ΔH, ΔS and ΔG values using a linear regression method and Microsoft Excel software (Microsoft Office 15) [26].

### 4.9. TPH2 Protein Quantification with Western Blot Analysis

The TPH2 levels were determined by means of Western blot analysis and as described earlier [25]. For TPH2 protein detection (at the 56 kDa level), a polyclonal rabbit antibody was used (1:1000 dilution, cat. # ab184505, Abcam, Cambridge, UK). The vinculin protein level was used as internal control, detected with a monoclonal rabbit antibody (1:2000, ab129002, Abcam) at 124 kDa. A goat anti-rabbit IgG antibody conjugated with horseradish peroxidase (secondary antibody; dilution 1:10,000, cat. # 31460, lot # SH253595, Invitrogen, Thermo Fisher Scientific, Waltham, MA, USA) was used for visualization. Protein bands were detected using a Fusion FX7-820 system (Vilber Lourmat, Collegien, France).

### 4.10. Assay of the Tph2 Gene mRNA Level

Total mRNA was extracted from the homogenate with ExtractRNA reagent (see Section 4.4.) according to the manufacturer’s protocol, treated with RNAase-free DNAase (Promega, Medisson, WI, USA) according to the manufacturer’s protocol and its concentration was diluted to the final concentration of 125 ng/μL. The cDNA was synthesized using a set of random hexanucleotide primers and R01 Kit according to the manufacturer’s protocol (Biolabmix, Novosibirsk, Russia). The mRNA level of target genes was assayed by qPCR using the set of selective primers (Table 1) and R401 Kit (Sintol, Moscow, Russia) according to the manufacturer’s protocol (95 °C 5 min; (95 °C, 15 s; annealing temperature, 60 s; 82 °C, 2 s; fluorescence registration) × 40 cycles). The threshold cycles were calibrated with a set of external standards containing 25, 50, 100, 200, 400, 800, 1600, 3200 and 6400 copies of genomic DNA extracted from C57BL/6 mouse liver. The gene expression was presented as a relative number of cDNA copies calculated on 100 copies of *Polr2a* cDNA as an internal standard [26].

### 4.11. Statistics

T_50_ and ΔG were calculated as described in [26]. All biochemical data were presented as the mean ± SEM and analyzed using one-way ANOVA. The body mass alterations were analyzed by repeated measure ANOVA with “Drug” as between and “Time” as within factors. Post hoc analyses were carried out using Fisher’s LSD multiple comparison test when appropriate. Statistical significance was set at *p* < 0.05.

## 5. Conclusions

In this study the effects of complex Fe(II) with tris(1-pyrazolyl)methane, [Fe(TPM)_2_]Cl_2_·2H_2_O on (1) activity and thermal stability in vitro and (2) activity in the brain (in vivo) of the mutant 447R form of TPH2 were investigated.

[Fe(TPM)_2_]Cl_2_·2H_2_O in the concentrations of 0.01, 0.05 and 0.1 mM significantly increased T_50_ and ΔG of the enzyme thermal denaturation in vitro. Moreover, this complex in concentrations of 0.05 and 0.1 mM increased the TPH2 activity in vitro. At the same time, the TPM ligand itself failed to alter the activity and thermal stability of TPH2 in vitro.

Intramuscular administration of 30 and 60 mg/kg of [Fe(TPM)_2_]Cl_2_·2H_2_O for 7 successive days to Balb/c mice with mutant TPH2 did not affect their body mass, but normalized the mutant TPH2 activity in the midbrain to the levels of the wild-type enzyme. The high dose of [Fe(TPM)_2_]Cl_2_·2H_2_O increased the TPH2 activity in the hippocampus.

This result is the first demonstration of the chaperone-like activity of [Fe(TPM)_2_]Cl_2_·2H_2_O. This complex seems to be a promising chemical for correction of genetic alterations in TPH2 and associated psychic disorders.

## Figures and Tables

**Table 1:** Sequences, annealing temperatures of the primers, and size of PCR products (amplicons).

Gene	Sequence	Annealing Temperatures, °C	Amplicon Size, bp
*Polr2a*	5′-TGTGACAACTCCATACAATGC-3′ 5′-CTCTCTTACTGAATTTGCGTACT-3′	60	194
*Tph2*	5′-CATTCCTCGCACAATTCCAGTCG-3′ 5′-AGTCTACATCCATCCCAACTGCTG-3′	62	239

## Data Availability

The original contributions presented in this study are included in the article/Supplementary Materials. Further inquiries can be directed to the corresponding author.

## Supplementary Materials

The following supporting information can be downloaded at: https://www.mdpi.com/article/10.3390/ijms27083411/s1.
