# Supplementary material for: Fe(II) with Tris(1-pyrazolyl)methane Complex Increases Thermal Stability In Vitro and Activity In Vivo of the Mutant 447R Form of Mouse Tryptophan Hydroxylase 2
Source: Int J Mol Sci. 2026 Apr 10;27(8):3411. doi: 10.3390/ijms27083411 (PMC13115991; doi:10.3390/ijms27083411)
Supplement: Supplementary file 1 [file ijms-27-03411-s001.zip › ijms-4206384-supplementary/Supplement_S1C (Legend to Supplements 1A-B).pdf]

Legend to TPH2 (Supplement S1A) and vinculin (Supplement S1B) blots

Band number from left to right (from 1 to 23)	Experimental groups:1-control, 2- 30 mg/kg, 3-60 mg/kg
1	1
2	2
3	3
4	1
5	2
6	3
7	1
8	2
9	3
10	1
11	2
12	3
13	1
14	2
15	3
16	1
17	2
18	3
19	1
20	2
21	3
22	1
23	2

Comment: the last two bands (22 and 23) were excluded from the final statistical analysis due to their low vinculin concentrations (see Supplement S1B)
