# Supplementary material for: Fe(II) with Tris(1-pyrazolyl)methane Complex Increases Thermal Stability In Vitro and Activity In Vivo of the Mutant 447R Form of Mouse Tryptophan Hydroxylase 2
Source: Int J Mol Sci. 2026 Apr 10;27(8):3411. doi: 10.3390/ijms27083411 (PMC13115991; doi:10.3390/ijms27083411)
Supplement: Supplementary file 1 [file ijms-27-03411-s001.zip › ijms-4206384-supplementary/Supplement_S2.pdf]

6.1. Male mice (n=24) with an average body mass of 27.9±0.3 g were divided into 3 groups of equal weight and number: (1) saline (control), (2) 30 mg/kg (corresponding to 3 mg iron/kg) and (3) 60 mg/kg (corresponding to 6 mg iron/kg). The drugs and saline were administered intraperitoneally for 7 days. On the 18th day, the animals were euthanized, the midbrain, hippocampus and hypothalamus were removed and stored at -80°C until TPH2 activity, 5-HT and 5-HIAA levels were assayed. No differences in body weight were found between these experimental groups ($F(2,21<1)$. In the midbrain, a high dose of the complex even decreased TPH2 activity, but did not affect the level of 5-HT and 5-HIAA (Table S1). In the hippocampus, $[Fe(TPM)_2]Cl_2$ did not affect TPH2 activity, the level of 5-HT and 5-HIAA (Table S1). In the hypothalamus, $[Fe(TPM)_2]Cl_2$ did not affect TPH2 activity but both doses of the drug reduced the level of 5-HT and 5-HIAA (Table S1).

Table S1. TPH2 activity, 5-HT and 5-HIAA levels in the midbrain hippocampus and hypothalamus of Balb/c mice after 7 ip injections of saline (control), 30 mg/kg and 60 mg/kg of $[Fe(TPM)_2]Cl_2$

	Control	30 mg/kg	60 mg/kg	p
Midbrain				
TPH2	33.2±2.0	28.1±2.4	25.2±1.4**	$F(2,20)=4.66$, $p=0.022$
5-HT	5.95±0.36	5.61±0.24	5.23±0.18	$F(2,20)=1.79$, $p=0.19$
5-HIAA	5.65±0.29	6.18±0.29	6.20±0.29	$F(2,20)=1.13$, $p=0.34$
Hippocampus				
TPH2	3.25±0.25	3.11±0.28	3.30±0.21	$F(2,21)<1$
5-HT	1.86±0.13	1.74±0.06	1.69±0.06	$F(2,21)=1.01$, $p=0.38$
5-HIAA	0.97±0.05	0.94±0.02	1.05±0.06	$F(2,21)=1.50$, $p=0.25$
Hypothalamus				
TPH2	10.40±0.64	10.07±0.50	10.16±0.56	$F(2,21)<1$
5-HT	3.93±0.07	3.48±0.08**	3.55±0.15*	$F(2,21)=5.18$, $p=0.015$
5-HIAA	1.29±0.0.06	1.09±0.03**	1.11±0.04**	$F(2,21)=6.86$, $p=0.005$

*$p<0.05$, **$p<0.01$ vs control.
